# Tunable Thermal Anisotropy Triggered by Quasi-Ballistic Heat Transport in WS_2_ Crystals

**DOI:** 10.1021/acs.nanolett.5c04514

**Published:** 2025-10-22

**Authors:** Kai Xu, Stefania Skorda, Peng Xiao, Emerson Coy, Xavier Cartoixà, Riccardo Rurali, Juan Sebastián Reparaz, Alexandros El Sachat

**Affiliations:** † Institut de Ciència de Materials de Barcelona, ICMAB-CSIC, Campus UAB, 08193 Bellaterra, Spain; ‡ Institute of Nanoscience and Nanotechnology, National Center for Scientific Research “Demokritos”, 15341 Agia Paraskevi, Athens, Greece; § Department of Applied Physics, National Technical University of Athens, Iroon Polytechniou 9 Zografou, 15780 Athens, Greece; ∥ Laboratoire Ondes et Matière d’Aquitaine (LOMA) - UMR 5798, CNRS, F-33400, Talence, France; ⊥ 529746 NanoBioMedical Centre, Adam Mickiewicz University, Wszechnicy Piastowskiej 3, Poznan 61-614, Poland; # Departament d’Enginyeria Electrònica, 16719 Universitat Autònoma de Barcelona, Bellaterra, 08193, Barcelona, Spain

**Keywords:** phonon transport, thermal conductivity anisotropy, frequency-domain thermoreflectance, WS_2_

## Abstract

We investigate the influence of temperature and film thickness on the anisotropic thermal conductivity tensor of multilayer single-crystal WS_2_ films of varying thickness (10 nm to 2.8 μm) across a wide temperature range (80–473 K). Experiments show that both in-plane (*k*_r_) and out-of-plane (*k*_*z*_) thermal conductivities increase with decreasing temperature, reaching, at 80 K in bulk WS_2_, values up to *k*_r_ ∼ 1000 W m^–1^ K^–1^ and *k*_*z*_ ∼ 13 W m^–1^ K^–1^. The thermal anisotropy ratio η = *k*_r_/*k*_*z*_ in bulk rises dramatically from 30 to 78 as the temperature decreases from 460 to 80 K, driven by the suppression of *k*_*z*_ due to phonon transport entering the quasi-ballistic regime. We further analyze the cumulative thermal conductivity as a function of phonon mean free path (MFP), showing that phonons with MFPs < 200 nm contribute to 70% of the total *k*_*z*_. This work provides fundamental insight into the interplay between dimensionality, temperature, and anisotropic phonon transport in two-dimensional materials, where thermal anisotropy can be strategically leveraged for performance optimization.

## Graphical abstract

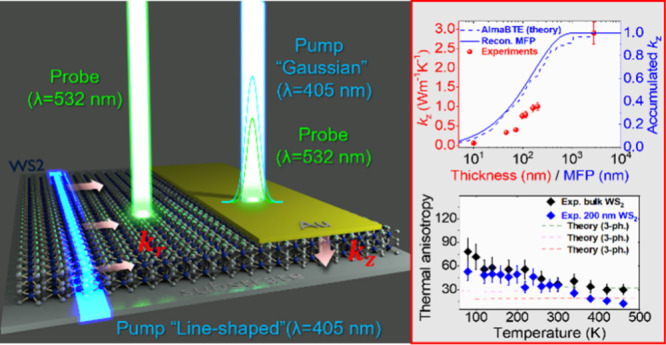

With the advent of increasingly sophisticated nanoscale electronic devices, two-dimensional (2D) materials have garnered significant interest due to their atomic-scale thickness, flexibility, high surface area, and exceptional anisotropic physical properties, showing already excellent performance in the fields of flexible electronics,^1^ optoelectronics,^2,3^ and nanoelectronics.^4^ As modern devices become increasingly compact and the demand for high-density integration continues to rise, effective thermal management has become essential. This necessity is particularly critical for devices based on 2D materials, which frequently operate across various temperature ranges and exhibit anisotropic heat dissipation characteristics. WS_2_ is among the most promising 2D semiconductors. Its tunable layer-dependent bandgap,^5^ ranging from 1.3 to 2.1 eV, carrier mobility (∼100 cm^2^ V^–1^ s^–1^),^6^ and remarkably high Seebeck coefficient (∼885 μV/K^7^) at 300 K find applications in nanoelectronics, including field-effect transistors,^8^ memristors,^9^ synaptic devices,^10^ and high-temperature thermoelectrics.^7^

Despite its considerable technological importance, its thermal conductivity tensor remains rather unexplored. At low temperatures, significant discrepancies persist between experiments and theory. Reported thermal conductivity values in the literature vary widely: for suspended films, the in-plane component of the thermal conductivity (*k*_r_) ranges from 28.5 to 63 W m^–1^ K^–1^, while for supported films, it spans from 15.4 to 32.8 W m^–1^ K^–1^.^11,12^ In contrast, the out-of-plane component of the thermal conductivity (*k*_*z*_) has been investigated only in a few experimental studies, focusing on synthetic bulk WS_2_, and its temperature dependence has been examined solely in the low temperature range (4–300 K).^13,14^ Reported room-temperature experimental values of *k*_*z*_ for bulk WS_2_ range from 3 to 1.7 W m^–1^ K^–1^ and decrease to approximately 0.35 W m^–1^ K^–1^ at 4 K,^14^ significantly diverging from theoretical predictions, which estimate values of 5.8 and 3.3 W m^–1^ K^–1^.^7,15^ Consequently, the calculated thermal anisotropy ratio (η = *k*_r_/*k*_*z*_) at low temperatures reaches unrealistically high values up to η ∼ 217 at 100 K.^14^ Additionally, the dependence of *k*_*z*_ on film thickness has yet to be experimentally investigated.

In this letter, we experimentally and numerically investigate the temperature dependence of the thermal conductivity tensor in crystalline WS_2_ films of varying thicknesses, enabling the determination of the temperature and thickness-dependent thermal anisotropy. To this end, we measure both *k*_*z*_ and *k*_r_ using complementary contactless techniques over a broad temperature range (80–473 K). Moreover, by systematically controlling the flake thickness, from bulk (2.8 μm) down to 10 nm, we access the quasi-ballistic transport regime and obtain the spectral mean free path (MFP) contributions to thermal conductivity. Lattice dynamics *ab initio* calculations corroborated our experimental findings and further elucidated the influence of film thickness and temperature on the thermal conductivity of WS_2_.

WS_2_ films were mechanically exfoliated from single crystals using a PDMS stamp and transferred onto SrTiO_3_ (STO) substrates following a reported method.^16^ The schematics of the WS_2_ crystal structure are shown in Figure 1a. The crystal, chemical, and morphological characterization of the samples studied by high-resolution scanning transmission electron microscopy (HR-STEM), X-ray photoelectron spectroscopy (XPS), atomic force microscopy (AFM), energy-dispersive X-ray spectroscopy (EDS), and angle-resolved polarized Raman scattering measurements indicate high-quality films free from polymer residues, as we show in Figure S1 of the Supporting Information (SI). Figure 1b displays a representative optical image of a 200 nm-thick WS_2_ flake. The Au-coated and uncoated areas were used to study *k*_*z*_ and *k*_r_, respectively. The inset of Figure 1b shows an AFM image of the STO/WS_2_ interface and its height profile. All film thicknesses are provided in Figure S1 of the SI. Figure 1c shows the Raman spectrum of the 200 nm-thick WS_2_ flake, where two Raman-active vibrational modes are observed at 356 cm^–1^ (E^1^_2g_) and 419.6 cm^–1^ (A_1g_), in agreement with previous reports.^17^ Room-temperature polar plots of Raman intensities for both modes at β = 0° and 90° are shown in Figures 1d,e. The HR-STEM image (Figure 1f) shows a honeycomb atomic arrangement, confirming the single-crystalline WS_2_ structure, while the Fourier transform pattern (Figure 1g) reveals a hexagonal lattice. Finally, XPS measurements shown in Figures 1(h–k) confirmed the expected chemical composition and stoichiometry of the WS_2_ flakes.

**Figure 1:**
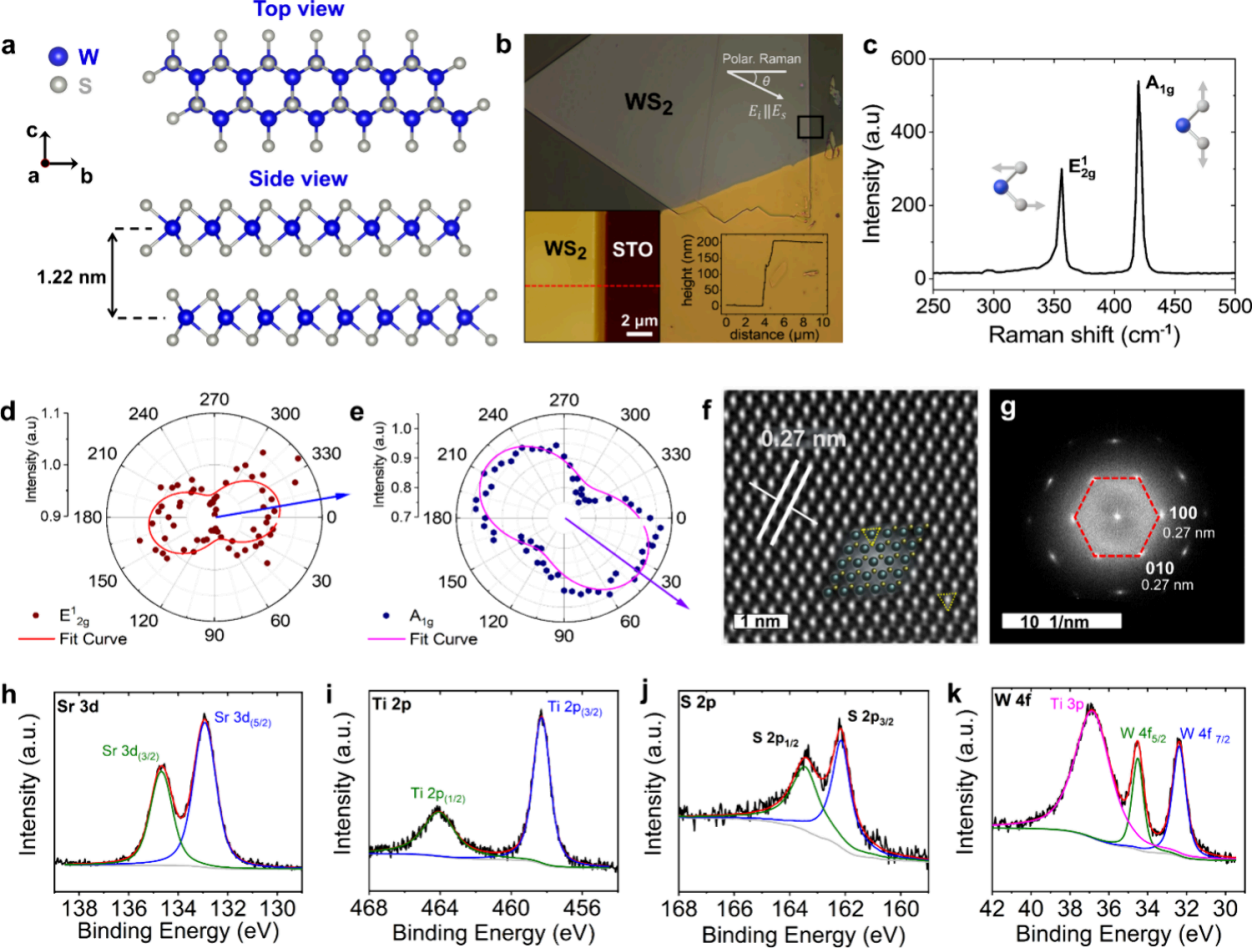
(a) Top and side views of the WS_2_ crystal structure. Each WS_2_ layer consists of three atomic sublayers, with tungsten (W) atoms (blue spheres) sandwiched between sulfur (S) atoms (gray spheres). (b) Representative optical image of a 200 nm-thick WS_2_ flake with a lateral size of approximately 300 μm. Insets show the AFM image of the region outlined by the black rectangle box and the corresponding height profile measured along the dashed red line in the topography image. (c) Representative Raman spectrum acquired using a 473 nm excitation wavelength and a 50× objective. The inset shows schematic arrows indicating the vibrational directions of W and S atoms. (d, e) Polar plots of Raman intensities for the 200 nm-thick WS_2_ film at 300 K. (f) HR-STEM image of the exfoliated WS_2_ flake and (g) the corresponding Fourier transform image showing the crystalline alignment. The interplanar spacing is labeled, and an atomic model of 2H-WS_2_ is superimposed on the image. Yellow dashed triangles highlight the arrangement of S atoms, characteristic of the 2H phase. We obtained in-plane lattice constants of *a* = *b* = 3.18 Å. (h–k) XPS data showing the binding energies of the Sr 3d, Ti 2p, S 2p, and W 4f core levels. The strong peaks at 32.4 eV (W 4f_7/2_) and 34.6 eV (W 4f_5/2_) depicted in Figure 1k confirmed the 2H phase of WS_2_, as previously reported.^18^

The *k*_*z*_ component of the tensor was measured using conventional frequency-domain thermoreflectance (FDTR),^19^ a noncontact technique that employs two focused laser beams with different wavelengths (405 and 532 nm) as the heater and thermometer, respectively. The output power of the heat source (405 nm) was harmonically modulated. On the other hand, *k*_r_ was characterized using a recently developed beam-offset FDTR method specifically designed to enhance the sensitivity to in-plane heat transport. This technique employs a one-dimensional (1D) heat source spatially offset from a point-like (0D) thermometer, both focused on the sample surface. The 1D heater geometry provides a slower spatial decay of the temperature field as compared to 0D heat sources, enabling signal detection at distances of tens of micrometers from the heat source.^20,21^ A 55 nm-thick gold transducer was deposited on selected regions of the WS_2_ flakes for the measurement of *k*_*z*_. In contrast, no transducer was used to obtain *k*_r_ since a sufficiently high thermoreflectance signal was obtained directly from the WS_2_ flake (see Section 2.2b of the SI). We recall that the use of a transducer is strictly necessary to obtain *k*_*z*_, since it limits the optical penetration depth of the laser, which substantially simplifies the data modeling procedure. In both experimental configurations (see Figure 2a), the measured physical quantity is the phase lag, *ϕ,* between the heater (subjected to harmonically modulated power) and the thermometer (which senses the temperature modulation induced by the pump).

**Figure 2:**
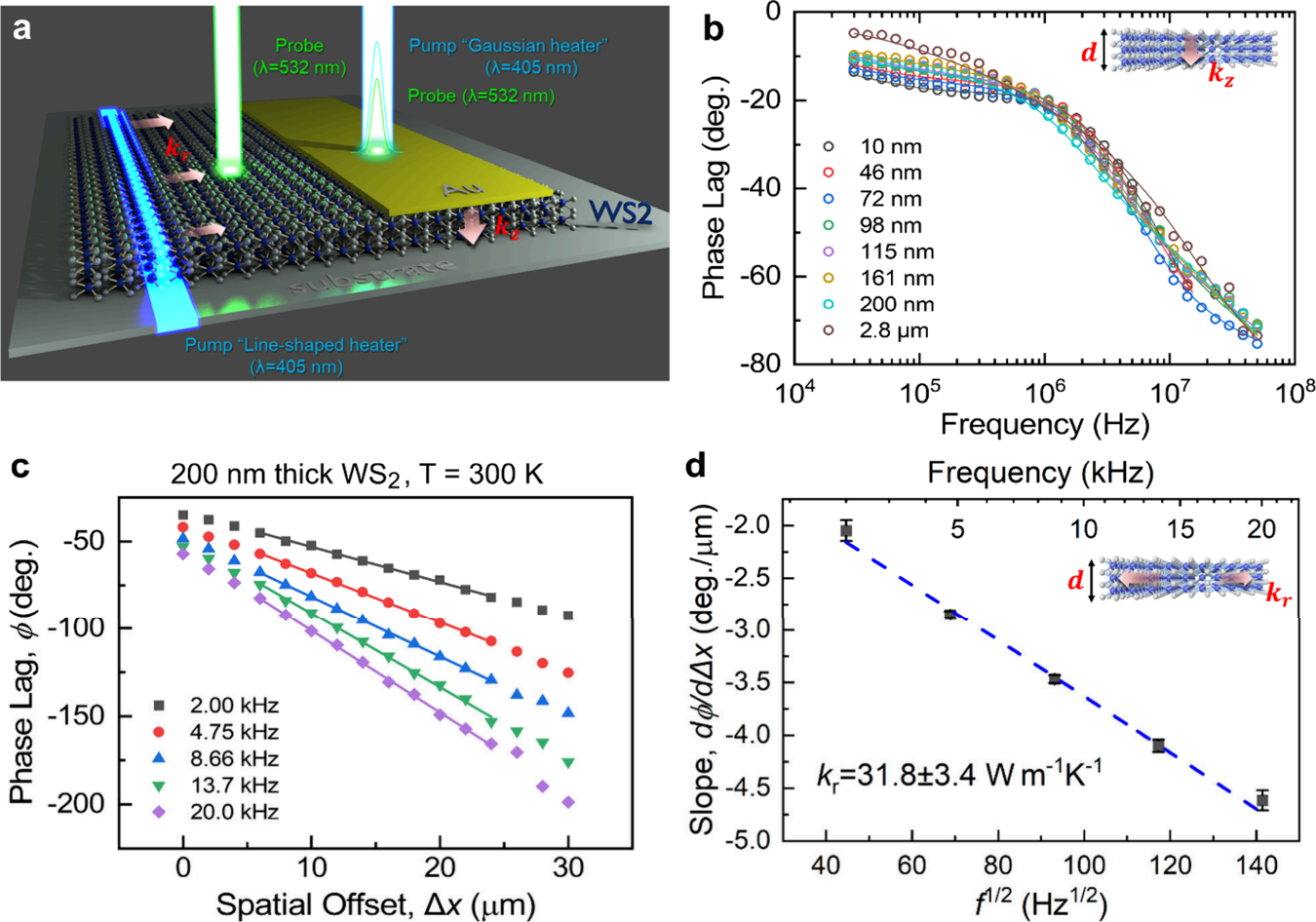
(a) Schematics of thermoreflectance experiments used to study out-of-plane and in-plane thermal transport. For in-plane thermal conductivity measurements (left), a line-shaped heater (blue laser) was applied directly onto the WS_2_ film, without the use of a metallic transducer, while the probe beam (green laser) was positioned at a controlled lateral offset, Δ*x*, from the pump. For out-of-plane thermal conductivity measurements (right), a conventional FDTR geometry was used, employing a focused Gaussian heat source. (b) Representative FDTR phase data measured for WS_2_ films of various thickness, along with corresponding model fits, across an excitation frequency range of 30 kHz to 50 MHz. (c) Phase lag as a function of the spatial offset for a 200 nm-thick WS_2_ flake at 300 K, measured at excitation frequencies between 2 kHz and 20 kHz. Solid lines represent linear fits to the data. (d) Slope obtained from the linear fits of ϕ vs Δ*x* for each frequency, plotted as a function of the inverse square root of the excitation frequency (*f* ^1/2^).

Figure 2b displays the phase lag curves as a function of the *f* measured at room temperature for all samples with different thickness. The solid lines represent the fits to the data points, which were used to extract *k*_*z*_. To simulate the response curves, we used a multilayer three-dimensional heat diffusion model, as described in Section 2.1 of the SI and reported elsewhere.^22–24^ The fitting parameters used within this framework are *k*_*z*_, the interfacial thermal conductance (ITC) Au/WS_2_ (*G*_1_), and the ITC WS_2_/STO substrate (*G*_2_). In Section 2.1 of the SI we describe in detail the followed step-by-step fitting methodology, showing for each thickness the model fitting curves (see Figure S2) and the extracted *k*_*z*_, *G*_1_, and *G*_2_ values with the corresponding fitting errors (*e*, the standard deviation between the experimental data and the values given by the fitted model) (see Table S1 of the SI). The calculated phase sensitivity coefficient to *G*_1_, *G*_2_, and *k*_*z*_ as a function of thickness and modulation frequency and all the parameters of the heat transfer model used in the sensitivity analysis are presented in Figure S3 and Table S2 of the SI. The temperature-dependent thermal conductivity of Au transducer and STO substrate was obtained through independent FDTR experiments on a precalibrated Si substrate, as well as confirmed through electrical conductivity measurements using the Wiedemann–Franz law (see Section 2.1b of the SI, Figure S5). The specific heat capacities of Au, STO, and WS_2_ were taken from previous works.^25–27^ All the details of the fitting analysis are provided in the SI. The *k*_r_ of the films was obtained using the relation^21,23^

$$\frac{\partial^{2}\phi }{\partial \Delta x\partial f^{0.5}}=\sqrt{\frac{C\rho \pi }{k_{r}}}$$

where ρ = 7500 kg·m^–3^ is the density^28^ and *C* = 256 J·kg^–1^·K^–1^ is the specific heat capacity^27^ of WS_2_ (our *ab initio* calculations yield 253 J·kg^–1^·K^–1^). Figures 2c,d show representative data used to extract *k*_r_. First, ϕ was measured as a function of the Δ*x* between the heater and thermometer lasers for several *f*. As shown in Figure 2c, ϕ exhibits a linear dependence on the Δ*x* for each *f*. Subsequently, the slope of each phase-vs-offset curve is determined and plotted as a function of *f* ^1/2^. The slope of this linear fit yields the *k*_r_ as shown in Figure 2d. A detailed discussion regarding the uncertainty estimation of the *k*_r_ is provided in Section 2.1a of the SI. Additionally, the measurements of *k*_r_ were also conducted by placing the probe beam (thermometer) on the gold-coated region to rule out any optical interference arising from optical excitation (see SI, Section 2.2c and Figure S6). These results clearly demonstrate that the presence or absence of the metallic transducer does not influence the determination of the *k*_r_ of the WS_2_ flakes. Moreover, this aspect has been extensively examined in our previous work.^21^

Figure 3a displays the experimental data corresponding to *k*_*z*_ (red symbols) for all the studied films. The uncertainty estimation of the *k*_*z*_ is discussed in Section 2.1a of the SI. We observe that *k*_*z*_ decreases by more than 1 order of magnitude by decreasing the film thickness from bulk down to 10 nm, with values ranging between 2.9 and 0.055 W m^–1^ K^–1^. The selected thicknesses span over 3 orders of magnitude, extending into the nanometer regime, with the specific purpose of investigating quasi-ballistic heat transport. This allows us to probe the spectral contributions of phonons with varying MFPs to the accumulated thermal conductivity of the thin films. Specifically, from the thickness dependence of *k*_*z*_, we obtain the accumulated thermal conductivity as a function of the phonon MFP, i.e., the percentage contribution to the total thermal conductivity of phonons with MFP smaller than a specific value. This approach, known as the reconstruction method, was initially proposed by Minnich.^29^ Within this approximation, the thermal conductivity of the thin films is expressed as *k*_*i*_ = ∫ *S*(*x*_*i*_)*f*(*Λ*_*ω*_)*dΛ*_*ω*_ where *k*_*i*_ (*k*_*z*_, in our case) is the thermal conductivity of each thin film with thickness *d*, *f*(*Λ*_*ω*_) is the differential MFP distribution function, *S*(*x*_*i*_) is the suppression function, and $x_{i}=\frac{\Lambda_{\omega }}{d}$ is the Reynolds number.

**Figure 3:**
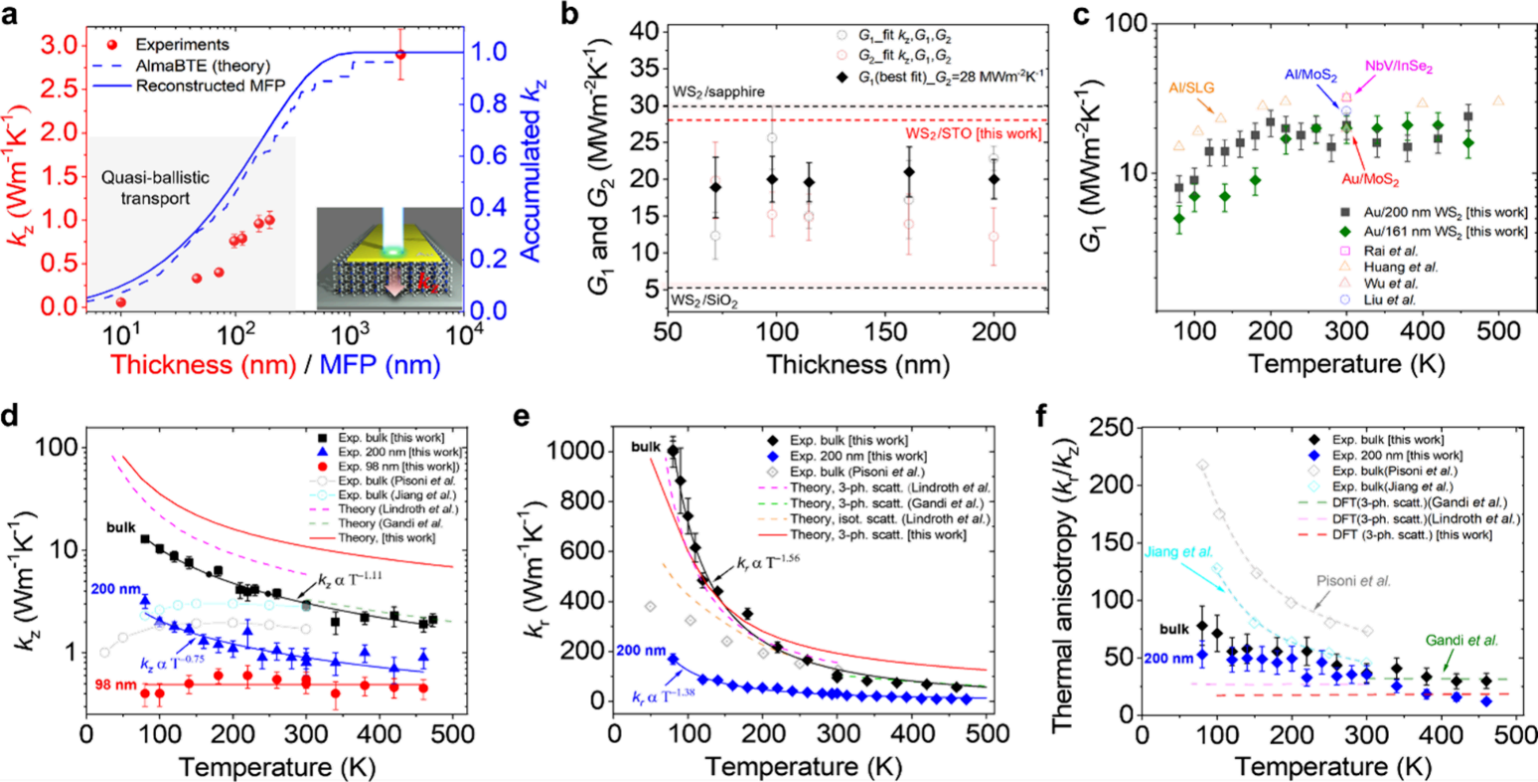
(a) Room-temperature *k*_*z*_ versus film thickness measured in exfoliated WS_2_ films (red solid symbols), the reconstructed thermal conductivity curve obtained from the experimental data (solid blue line), and our *ab initio* calculations (dashed blue line) based on the solution of the phonon Boltzmann transport equation (BTE). (b) Interface thermal conductance between Au/WS_2_ (*G*_1_) and WS_2_/STO (*G*_2_) at 300 K versus film thickness. Shaded circles show the extracted *G*_1_ and *G*_2_, respectively, by simultaneously fitting all the unknown parameters. Solid black triangles show the best fit values of *G*_1_ for *G*_2_ = 28 MW m^–2^ K^–1^. The dashed black lines show previous *G*_2_ values obtained in WS_2_/sapphire^30^ and WS_2_/SiO_2_ ^31^ substrates. (c) Temperature dependence of the *G*_1_ obtained in Au/WS_2_ interfaces (solid symbols) compared to previous measurements (open symbols) in Al/single-layer graphene (SLG),^32^ Au/MoS_2_,^33^ Al/MoS_2_,^34^ and NbV/InSe_2_.^35^ (d) Temperature dependence of the *k*_*z*_ measured in bulk and 200 and 98 nm-thick WS_2_ films (solid symbols) compared to previously reported experimental values (open circles) in bulk WS_2_.^13,14^ (e) Temperature dependence of the *k*_r_ measured in bulk and 200 nm-thick WS_2_ films (solid symbols) compared to previous measurements (open triangles) in bulk WS_2_.^14^ (f) Temperature dependence of the anisotropic thermal conductivity ratio measured in this work (solid symbols). Open symbols show previously reported experimental η values.^13,14^ In (d), (e), and (f) we plot our first-principles calculations (solid and dashed red lines) and previous DFT calculations^7,15^ (see dashed pink and green lines). Experimental data and theoretical curves plotted in (d), (e), and (f) reproduced from ref 7, Copyright 2014 American Chemical Society, and with permission from refs 13, Copyright 2017 [WILEY-VCH Verlag GmbH & Co. KGaA, Weinheim],^14^, Copyright 2016 [Elsevier], and^15^, Copyright 2016 [American Physical Society].

The FDTR experiments yield eight independent measurements for *k*_*z*_(*d*), corresponding to each of the investigated thin films with different thicknesses. In practice, each measured value is reduced by an amount that reflects the suppressed contribution of phonon modes with MFPs comparable to *d*. For a specific heating geometry, a heat flux suppression function *S*(*Λ*_*ω*_, *d*) describes how phonon modes with MFP *Λ*_*ω*_ are attenuated as a function of the film thickness. To obtain the accumulated thermal conductivity function, *F*(Λ_ω_) = ∫_0_^∞^ *f*(Λ′)*dΛ*′, the inverse problem is solved using convex optimization. We note that the main advantage of performing this transformation is that the resulting accumulated thermal conductivity versus MFP is an intrinsic property of the material; consequently, it does not depend on the specific experimental conditions. Figure 3a displays the reconstructed thermal conductivity curve (solid blue line), obtained from the experimental data using the reconstruction procedure described above. For comparison, we plot our results from *ab initio* calculations based on the solution of the phonon Boltzmann transport equation (BTE), demonstrating an excellent agreement with the experimental measurements. For example, we find that phonons with MFPs < 200 nm account for approximately 70% of the bulk cross-plane thermal conductivity, whereas those with MFPs < 10 nm contribute less than 30%. This analysis provides valuable insights into the relevant length scales of phonon MFPs that must be controlled to effectively tailor the *k*_*z*_ of WS_2_. Figures 3b,c display the extracted *G*_1_ and *G*_2_ for each film thickness (see fitting details in Section 2.1 of the SI).

The temperature dependence of *k*_*z*_ for WS_2_ flakes with thicknesses of 98 nm, 200 nm, and 2.8 μm is shown in Figure 3d. The thickest samples (200 nm and 2.8 μm) exhibit a temperature dependence that aligns with the empirical relation *k* ∝ *T* ^–*n*^, commonly observed in semiconductors, where the exponent *n* is influenced by the dominant phonon scattering mechanisms, such as Umklapp, boundary, and impurity scattering.^36^ Fitting the experimental data yields *n* = 1.11 and *n =* 0.75 for the 2.8 μm and 200 nm-thick samples, respectively. In contrast, the 98 nm-thick flake shows an almost negligible temperature dependence as phonon-boundary scattering becomes the predominant scattering mechanism. This trend suggests that thinner flakes are expected to exhibit similarly weak temperature dependence. The theoretical results exhibit a considerable dispersion and, in general, do not provide quantitative predictions of *k*_*z*_, at least not as good as for *k*_r_, as we discuss below. This behavior can be ascribed to the subtleties involved in an accurate modeling of interlayer vdW interactions. We used the semiempirical DFT-D2 method of Grimme^37^ and obtained a *c* lattice vector of 11.56 Å, shorter than the experimental value.^38^ This is most likely the reason for the overstatement of *k*_*z*_. Lindroth and Erhart^15^ used vdW-DF,^39^ a fully *ab initio* functional to account for vdW interactions. Their lattice parameter is closer to our experimental values, but they used a rather small 3 × 3 × 1 supercell, and their cutoff for the calculation of third-order force constants is underconverged, according to our tests (see the SI). Finally, Gandi and Schwingenschlögl^7^ achieved the best agreement with the experimental data using DFT-D3,^40^ a vdW functional similar to ours; the supercell and cutoff for anharmonic interactions are slightly larger than those of ref 15, but the latter appears to be still undercoverged.

The temperature dependence becomes stronger in the in-plane direction, as shown in Figure 3e. We obtained *n* = 1.56 and *n =* 1.38 for the 2.8 μm and 200 nm-thick samples, respectively, which are higher compared to the values observed for the out-of-plane direction in Figure 3d. This result is explained considering that within the in-plane directions, the influence of phonon boundary scattering is not as strong as compared to the out-of-plane case. Specifically, for bulk WS_2_, *k*_r_ decreases from 1006.7 ± 35.5 to 56.7 ± 5.8 W m^–1^ K^–1^ (i.e., more than 1 order of magnitude) as temperature increases from 80 to 460 K, in excellent agreement with previous first-principles calculations^7,15^ and with those performed in this work. Similarly, for the same temperature range in the 200 nm-thick flake, *k*_r_ decreases from 169.7 ± 20.3 W m^–1^ K^–1^ (80 K) to 7.2 ± 0.4 W m^–1^ K^–1^ (473 K). Both samples exhibit a comparable reduction in *k*_r_, with values decreasing by approximately a factor of 20 in the temperature range studied. Measurements in previous works^13,14^ reported much lower values in bulk WS_2_ for both *k*_r_ and *k*_*z*_.

Figure 3f presents η values extracted from the experimental data shown in Figures 3d,e for the 2.8 μm (bulk) and 200 nm thick WS_2_ films. In the bulk sample, we observe a significant increase in η with decreasing temperature, rising by more than a factor of 2 from 460 to 80 K. This behavior likely arises from boundary scattering effects, which are more pronounced along the out-of-plane direction compared to the in-plane direction. Specifically, boundary scattering imposes stronger restrictions on phonon transport along the out-of-plane direction, reflected in the distinct temperature dependence exponents observed for *k*_*z*_ and *k*_r_. Consequently, as temperature decreases, *k*_r_ increases more rapidly than *k*_*z*_, enhancing the anisotropy factor. The 200 nm thick film exhibits considerably lower values across the entire measured temperature range; that is, η increases from 12 (460 K) up to 53 (80 K). Notably, the 200 nm sample shows a more pronounced variation in η (approximately a 4-fold increase from high to low temperatures) compared to the bulk sample, which shows only a 2-fold variation. This enhanced sensitivity originates from enhanced boundary scattering in thinner samples, where its surface plays a comparatively more dominant role. The obtained thermal anisotropy, η = 36, for the bulk sample at 300 K aligns closely with previous calculations by Gandi et al.^7^ (η ≈ 32) and Lindroth et al.^15^ (η ≈ 27).

We note that previous experiments^13,14^ reported significantly higher anisotropy factors at room temperature for bulk WS_2_ of η = 70 and η = 45, respectively. This discrepancy becomes larger at low temperatures primarily due to the substantially lower measured *k*_*z*_ values arising most likely from differences in sample quality, measurement techniques, or interfacial conditions. Specifically, in the work of Pisoni et al.,^14^ the samples have been fabricated using the chemical vapor transport (CVT) method, while a silver paste has been used to pile different WS_2_ crystals into stacked structures, introducing additional thermal resistances with this way. The authors of this work also discussed the possibility that impurities present in the starting materials for synthesis caused doping in their crystals. Similarly, in the work of Jiang et al.^13^ the samples were prepared using the low-pressure vapor transport method. In contrast, our films were prepared by mechanical exfoliation from high-quality, single-crystalline bulk crystals, followed by transfer using a viscoelastic stamp onto substrates. This approach preserves the intrinsic lattice structure of WS_2_ and ensures pristine, undoped interfaces, both of which are essential for reliable thermal transport measurements. Finally, additional factors, such as interfacial roughness or uncertainties related to spot size measurements, may also contribute to the observed discrepancies. Our experimental thermal anisotropy values match closely with the theoretical anisotropy curves shown in Figure 3f, reinforcing the consistency and reliability of our findings.

In summary, we investigated the anisotropic thermal conductivity of bulk and thin WS_2_ films versus temperature experimentally and numerically. Bulk WS_2_ exhibits an exceptionally high *k*_r_ consistent with theory and tunable thermal anisotropy. Thin films exhibit suppressed thermal anisotropy and much lower *k*_*z*_. The latter becomes insensitive to temperature as the thickness decreases due to increased boundary scattering. The systematic variation of film thickness enables us to probe the quasi-ballistic phonon transport regime and quantify the spectral contributions of phonon MFP to the cross-plane thermal conductivity, showing that phonons with MFPs < 200 nm contribute to 70% of the total *k*_*z*_. In contrast, the large values observed for *k*_r_ indicate that thermal phonons propagating in the in-plane direction have much larger MFPs. Our experimental and theoretical results advance understanding of heat transport in anisotropic 2D materials and suggest strategies for thermal management in electronic, optoelectronic, and thermoelectric devices. By enabling directional heat flow and mitigating hot spots, WS_2_ can function as both an active material and heat spreader, supporting efficient, thermally stable 2D device operation across varying temperatures.

## Supplementary Material



## Data Availability

The data that support the findings of this study are available from the corresponding author upon reasonable request.

## Notes

Optical images, EDS and AFM data, FDTR sensitivity analysis, conventional and beam-offset FDTR measurements, fitting and uncertainty analysis, and combined experimental and computational methods (PDF)
