# Supplementary material for: Tunable Thermal Anisotropy Triggered by Quasi-Ballistic Heat Transport in WS2 Crystals
Source: Nano Lett. 2025 Oct 22;25(44):16006–12. doi: 10.1021/acs.nanolett.5c04514 (PMC12593381; doi:10.1021/acs.nanolett.5c04514)
Supplement: Supplementary file 1 [file nl5c04514_si_001.pdf]

Supporting Information:

Tunable thermal anisotropy triggered by quasi-ballistic heat transport in WS_2 crystals

Kai Xu,[1,2] Stefania Skorda,[2,3] Peng Xiao,[4] Emerson Coy,[5] Xavier Cartoixà,[6] Riccardo Rurali,[1] Juan Sebastián Reparaz[1] and Alexandros El Sachat[2]**

[1]Institut de Ciència de Materials de Barcelona, ICMAB-CSIC, Campus UAB, 08193 Bellaterra, Spain

[2]Institute of Nanoscience and Nanotechnology, National Center for Scientific Research "Demokritos," 15341 Agia Paraskevi, Athens, Greece

[3]Department of Applied Physics, National Technical University of Athens, Iroon Polytechniou 9 Zografou, 15780 Athens, Greece

[4]Laboratoire Ondes et Matière d'Aquitaine (LOMA) - UMR 5798, CNRS, F-33400, Talence, France

[5]NanoBioMedical Centre, Adam Mickiewicz University, Wszechnicy Piastowskiej 3, Poznan 61-614, Poland

[6]Departament d'Enginyeria Electrònica, Universitat Autònoma de Barcelona, Bellaterra, 08193, Barcelona, Spain

1. Optical images, AFM and EDS measurements of exfoliated WS_2 flakes

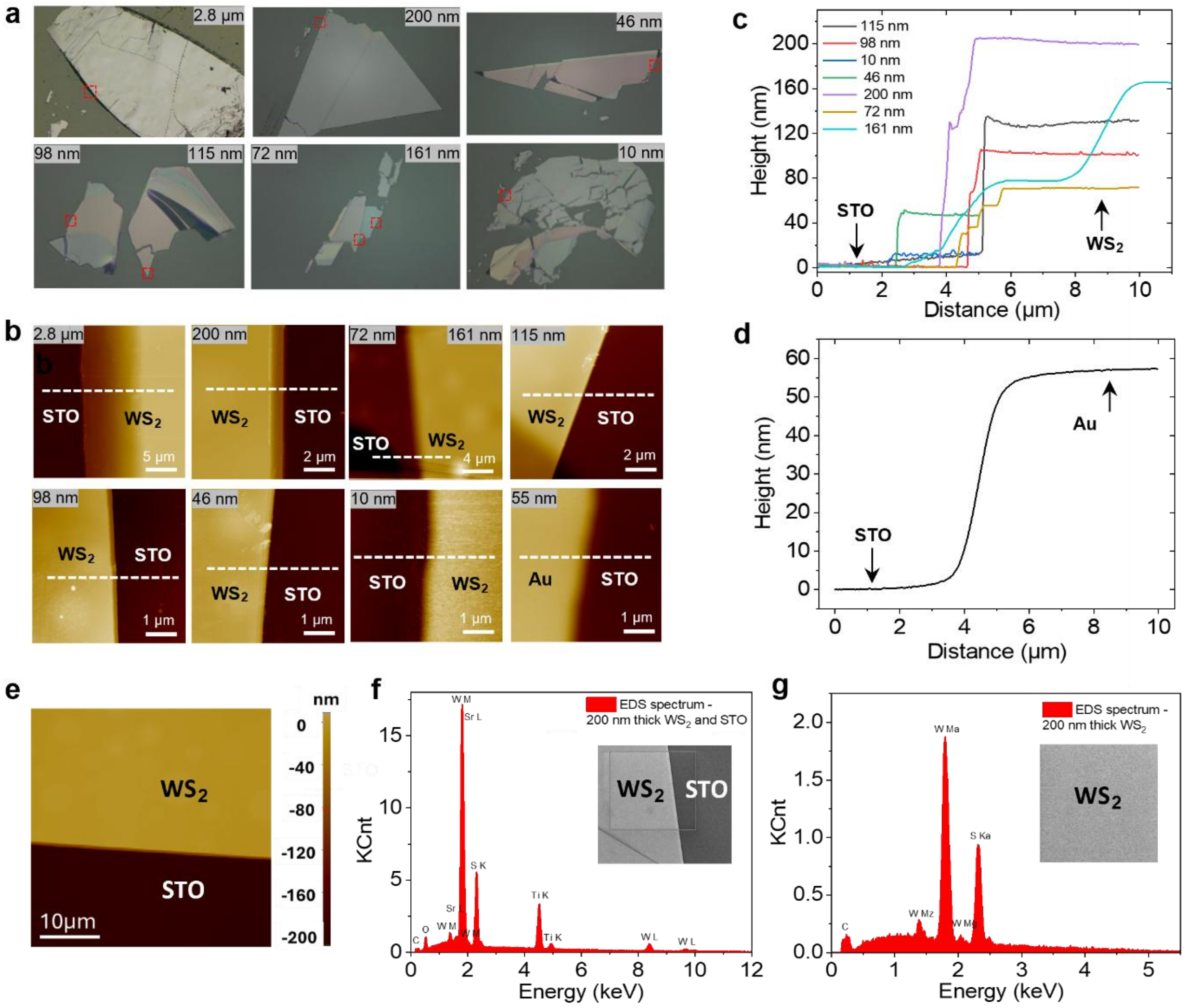

Figure S1. (a) Optical images of exfoliated WS_2 flakes of different thickness on STO substrates using pre-cleaned PDMS with soft O_2 plasma to improve mold adhesion and suppress oligomer migration during curing, enhancing surface cleanliness. (b) Atomic force microscopy (AFM) images showing the interfaces between WS_2 films and the STO substrate and (c) the corresponding topography profiles. The dashed squared boxes in (a) indicate the selected areas for the AFM maps. (d) Topography profile between Au and STO substrate. (e) High-resolution

AFM image of the 200 nm thick WS_2 revealing a clean surface, Energy-dispersive X-ray spectroscopy (EDS) spectra from a (f) 40 x 40 μm^2 area including the STO and the WS_2 flake (see squared region of the SEM image in (f)) and (g) a 25x25 μm^2 area including only the WS_2 flake. The absence of the characteristic oxygen peak at (0.5 keV) associated with PDMS[1] suggests that no detectable polymer residue remains on the surface.

2. FDTR experimental data processing, sensitivity analysis and uncertainty estimation

In Figure 3b of the main text we show the extracted G_1 and G_2 for each film thickness at 300 K, obtained using the fitting procedure outlined in Section 2.1 below. For G_2, we used an average value of ~28 $MWm^{-2}K^{-1}$ determined from fitting samples with thicknesses between 47 and 200 nm. Assuming that G_2 remains approximately constant in the 71 and 200 nm film thickness range, we estimated G_1 (solid black triangles in Figure 3b) and compared these results with those obtained from simultaneous fitting of k_z, G_1 and G_2 (open black and red circles in Figure 3b). Both methods yield consistent results, confirming the reliability of the fitting strategies employed. Moreover, the step-by-step fitting analysis (see Table S1 below) shows that variations in G_1 have negligible impact on the extracted k_z values of the WS_2 films.

2.1 Conventional FDTR to obtain out-of-plane thermal conductivity and interface thermal conductance

Figure S2 shows the FDTR experimental data of thickness series samples and corresponding fitting curves. The evolution of cross-plane thermal conductivity of WS_2 (k_z), interface thermal conductance of Au/WS_2 (G_1) and WS_2/STO (G_2), and the fitting residuals (e, the standard

deviation between the experimental data and the values given by the fitted model) obtained by each fitting step are summarized in Table S1.

Step-by-step fitting methodology: To fit the FDTR data across the thickness series, we began with simple models and gradually increased their complexity by stepwise incorporation of thermal boundary resistances ($G_1=1/R_1$ and $G_2=1/R_2$). This approach validated the robustness of the thermal conductivity fits while also revealing differences in the sensitivity of the fits to the presence of interface thermal conductance's across samples of varying thickness.

The detailed fitting strategy is outlined as follows: In **Step 1**, the two thermal boundary resistances were excluded, and only k_z was fitted. This simplified model allowed us to explore a broad parameter range and estimate the order of magnitude of k_z. However, e remained high for most samples, except for the 47 nm and 72 nm samples. In **Step 2**, G_1 was added as a second fitting parameter. This improved the fit for the thicker samples, but not for the three thinnest, where the errors remained high or increased slightly. Their G_1 values were not considered representative (pink filled values in Table S1). For the rest, the fitted G_1 values were retained as inputs for Step 3 (green filled values in Table S1).

Assuming comparable interfaces due to the identical materials and fabrication process, we assigned the average G_1 value (20 MW m^{-2} K^{-1}) to the three thin samples in **Step 3**. At this stage, G_2 was introduced as a fitting parameter, which reduced errors in most cases, although the bulk sample exhibited an increase. Thus, we excluded the G_2 for the bulk sample obtained in this step. In **Step 4**, the average value of G_2 value obtained from Step 3 was fixed, and k_z and G_1 were refitted. This adjustment had little effect on most samples but reduced the error for the bulk sample. Finally, in **Step 5**, all three parameters were fitted simultaneously. This approach

yielded lower overall errors, while the k_z values remained relatively stable across most cases, confirming the robustness of the fit. As shown in Figure S2, the film thicknesses are extremely relative to the thermal penetration depth defined by the measurement frequency range and heat transfer model. Consequently, the 10 nm sample and the bulk sample are insensitive to G_1 and G_2, respectively. These two samples were therefore excluded from the full fitting procedure, with their fitting processes ending at **Step 3** and **Step 4**, respectively.

As evidenced by the fitting process, a key challenge in the data analysis is the wide variation in WS_2 flake thicknesses, which results in differing parameter sensitivities across the same frequency range. To ensure the reliability of the fitted results, a sensitivity analysis was conducted in a way like A. Schmidt *et al.*[2] to assess the influence of each parameter on the phase and confirm the robustness of the extracted thermal properties. The parameters of the heat transfer model used in the sensitivity analysis are given in Table S2.

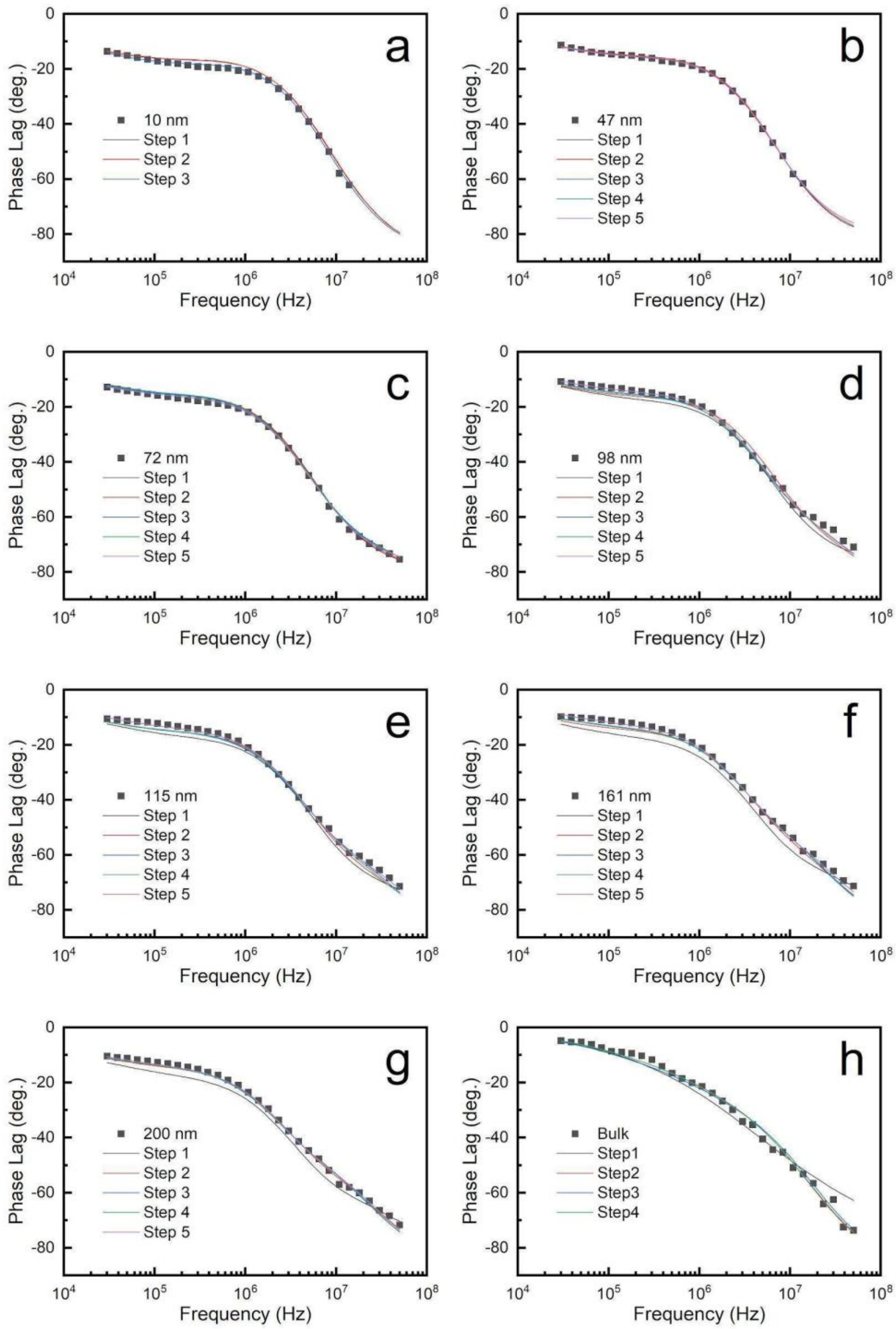

Figure S2. FDTR phase data measured for WS_2 flakes with thicknesses ranging from 10 nm to 2.8 μm (bulk), and the corresponding step-by-step model fitting curves.

Table S1. The key parameters in the step-by-step fitting of FDTR results

Sample	Step1 (fit k_z no $G1$, G_2)		Step2 (fit k_z, $G1$ no $G2$)			Step3 (fit k_z, $G2$ fix $G1$)				Step4 (fit k_z, $G1$ fix $G2$)				Step5 (fit k_z, $G1$, $G2$)			
	k_z (W×m^{-1}K^{-1})	e (Fitting residual)	k_z (W×m^{-1}K^{-1})	$G1$ (MW×m^{-2}K^{-1})	e (Fitting residual)	k_z (W×m^{-1}K^{-1})	$G1$ (MW×m^{-2}K^{-1})	$G2$ (MW×m^{-2}K^{-1})	e (Fitting residual)	k_z (W×m^{-1}K^{-1})	$G1$ (MW×m^{-2}K^{-1})	$G2$ (MW×m^{-2}K^{-1})	e (Fitting residual)	k_z (W×m^{-1}K^{-1})	$G1$ (MW×m^{-2}K^{-1})	$G2$ (MW×m^{-2}K^{-1})	e (Fitting residual)
10 nm	0.05	0.312594	0.05	260	0.315346	0.055	20	25	0.179986	-	-	-	-	-	-	-	-
47 nm	0.23	0.079351	0.24	50	0.102279	0.3	20	19	0.074468	0.3	40	28	0.074744	0.3	12	20	0.070273
72 nm	0.33	0.198201	0.32	55	0.188049	0.38	20	34	0.164042	0.39	19	28	0.198249	0.4	30	35	0.175242
98 nm	0.57	0.493011	0.7	15	0.365710	0.8	15	34	0.246394	0.76	20	28	0.284840	0.82	26	15	0.246321
115 nm	0.62	0.515897	0.62	20	0.319763	0.88	20	13	0.281363	0.78	20	28	0.287152	0.83	14	15	0.283306
161 nm	0.78	0.706490	0.8	24	0.347111	1	24	28	0.286101	0.98	21	28	0.281913	0.92	17	13	0.280512
200 nm	0.96	0.571093	0.88	17	0.230133	0.96	17	45	0.230330	1	20	28	0.232852	0.94	23	12	0.216424
Bulk	4.3	0.696963	2.8	22	0.309361	2.8	22	65	0.357638	2.8	12	28	0.309893	-	-	-	-

Table S2. Parameters for FDTR measurements sensitivity calculation of WS_2

Sample	Spot Size (μm)		Au Transducer				WS_2				STO Substrate				Thermal Boundary Conductance	
	Pump	Probe	Thickness (nm)	Thermal conductivity (W×m^{-1}K^{-1})	Heat capacity (J×kg^{-1}K^{-1})	Density (kg×m^{-3})	Thickness (nm)	Thermal conductivity (W×m^{-1}K^{-1})	Heat capacity (J×kg^{-1}K^{-1})	Density (kg×m^{-3})	Thickness (mm)	Thermal conductivity (W×m^{-1}K^{-1})	Heat capacity (J×kg^{-1}K^{-1})	Density (kg×m^{-3})	G1 (MW×m^{-2}K^{-1})	G2 (MW×m^{-2}K^{-1})
10 nm	4	4.5	55	230	128	19400	10	0.055	260	7500	1	8	520	5150	20	25
47 nm	4	4.5	55	230	128	19400	47	0.3	260	7500	1	8	520	5150	12	20
72 nm	4	4.5	55	230	128	19400	72	0.4	260	7500	1	8	520	5150	30	35
98 nm	4	4.5	55	230	128	19400	98	0.82	260	7500	1	8	520	5150	26	15
115 nm	4	4.5	55	230	128	19400	115	0.83	260	7500	1	8	520	5150	14	14
161 nm	4	4.5	55	230	128	19400	161	0.92	260	7500	1	8	520	5150	17	13
200 nm	4	4.5	55	230	128	19400	200	0.94	260	7500	1	8	520	5150	23	12
Bulk	4	4.5	55	230	128	19400	2800	2.8*	260	7500	1	8	520	5150	12	28

* WS_2 layers are considered as isotropic for the sensitivity calculation except for the bulk, an anisotropic ratio of 35 is set for the bulk samples.

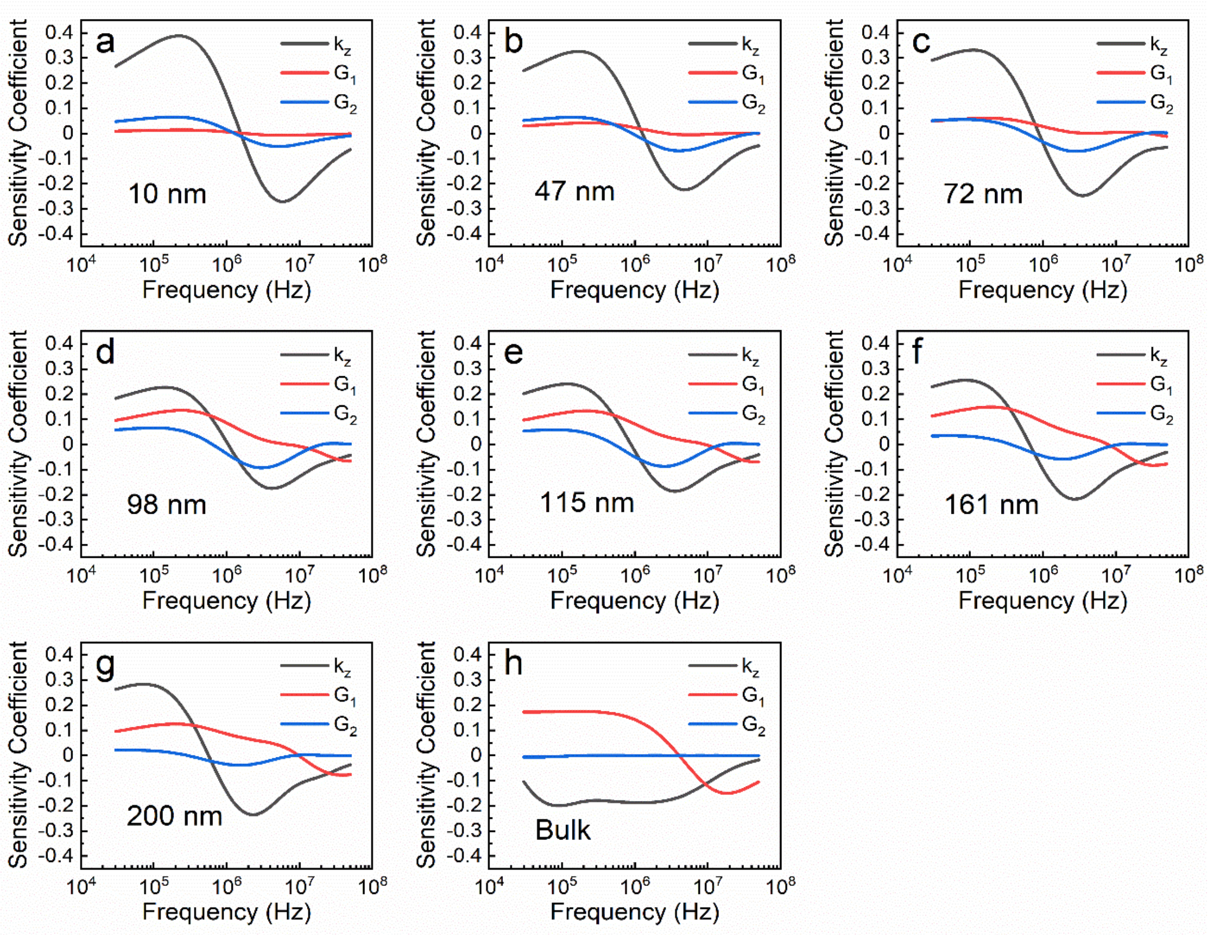

Figure S3. Calculated phase sensitivity coefficient ($S = \frac{x}{\phi}\frac{\partial \phi}{\partial x}$, in which x represents the value of certain parameters and ϕ is the phase lag) to different parameters G_1, G_2 and k_z, as a function of thickness and modulation frequency for the case of Au/WS_2/STO stacks.

As shown in Figure S3, under our experimental conditions, the thermal conductivity of WS_2 remains a sensitive parameter across all tested thicknesses. Except for the bulk sample, the phase sensitivity of thermal conductivity in the other samples shows similar behavior. A sign reversal occurs around 1 MHz, and this reversal frequency becomes lower as the sample thickness increases. This sign reversal is observed because as the frequency increases, the thermal penetration depth becomes smaller than the thickness of the structure on the substrate

(gold transducer and WS_2 flake). For the bulk sample, no reversal was seen within the measured frequency range, suggesting that the thermal penetration depth within the measurement frequency range is smaller than the sample thickness. The fitting also reveals sensitivity to both G_1 and G_2, with the sensitivity to G_1 increasing and to G_2 decreasing as the WS_2 thickness increases. For the 2.8 μm-thick sample, the heat flow does not significantly modulate across G_2, making it infeasible to accurately extract G_2 through fitting. To address this, we used the G_2 value obtained from thinner samples as a fixed input parameter during fitting of the 2.8 μm sample. Likewise, the fixed G_1 value for the 10 nm sample was taken from the average value of other samples as explained above.

2.1a Determination of the out-of-lane and in-plane experimental uncertainty

Out-of-plane thermal conductivity uncertainty

Combining the above sensitivity analysis results and the uncertainty of the measured phase (σ_ϕ), we estimated the error of the fitted thermal conductivity. To simplify, assuming the phase noise at each frequency point, i , is known and uncorrelated, the relative uncertainty in k can be derived from standard least-squares error propagation, giving:

$$\frac{u(k)}{k} = \sqrt{Var(ln\, k\,)} \approx \frac{1}{\sqrt{\sum_{i=1}^{N} \; S_k(f_i)^2 \sigma_{\phi,i}^2}}$$

This equation follows from the principle that the variance of a fitted parameter is inversely proportional to the squared signal-to-noise ratio of the sensitivity-weighted data. If the phase noise is approximately constant across frequencies ($\sigma_{\phi,i} = \sigma_\phi$), the expression simplifies to:

$$\frac{u(k)}{k} \approx \frac{\sigma_\phi}{\sqrt{\sum_{i=1}^{N} \; S_k(f_i)^2}}$$

which for the case of N measurements with uniform phase noise and roughly constant sensitivity, we can derive a very simple and practical approximation for the uncertainty in thermal conductivity:

$$\frac{u(k)}{k} \approx \frac{\sigma_\phi}{|S_k|\sqrt{N}}$$

As an example, considering that in all our experiments $N = 30$ according to the sensitivity curves present in Figure S3 and fitting residual e in Table S1, we obtain for a 72 nm thick sample, $S \approx 0.2$. Since the standard deviation of the actual experimental data is not directly known, we assume it to be equal to the fitting residual (e.g., $\sigma_\phi = e \approx 0.175$), which is a common empirical approximation for estimating the uncertainty of fitting parameters. The standard deviation of thermal conductivity can be estimated as $\frac{u(k)}{k}(72\,nm) \approx \frac{0.175}{\sqrt{30}\times 0.2} \approx 0.16 = 16\%$.

Figure S4 presents the FDTR data fitting results for two samples with different thicknesses. To further verify the rationality of the error estimation, we generated phase profiles with a ±25% output parameter error based on the heat transfer model, as shown in Figure S4. In Figure S4(a), we show the error bands of the fitting curves for the 72 nm-thick WS_2 sample, considering a ±25% variation in k_r and G_2. Since G_1 does not exhibit significant sensitivity in the measured frequency range, no fitting error band is provided for it in the plot. It can be observed that both parameters exhibit sensitivity to the phase lag fitting across the entire measurement frequency range. In particular, the phase curves corresponding to k_r ±25% fully enclose the measured data points. Under the assumption of monotonicity, this suggests that the fitting error is lower than ±25%. We estimated that the error was 16%, which is consistent with the trend shown in the figure. Notably, the upper and lower error bounds for both k_r and G_2 intersect around 1 MHz,

which closely corresponds to the frequency at which the sign of the calculated sensitivity curve (shown in Figure S3) changes from positive to negative. Similarly, Figure S4(b) presents the fitting of the measured data for the 2.8 μm-thick sample, along with the fitting error bands for k_r and the interfacial thermal conductance G_1, based on a ±25% variation. The frequency-dependent changes in the width of the error bands are also consistent with the calculated sensitivity of the corresponding parameters. Based on the parameter evolution during the fitting steps and the sensitivity analysis near the output values, the out-of-plane thermal conductivity of WS_2 extracted from the FDTR model is representative, and the thermal boundary conductance's (G_1 and G_2) obtained from intermediate-thickness samples also serve as useful references.

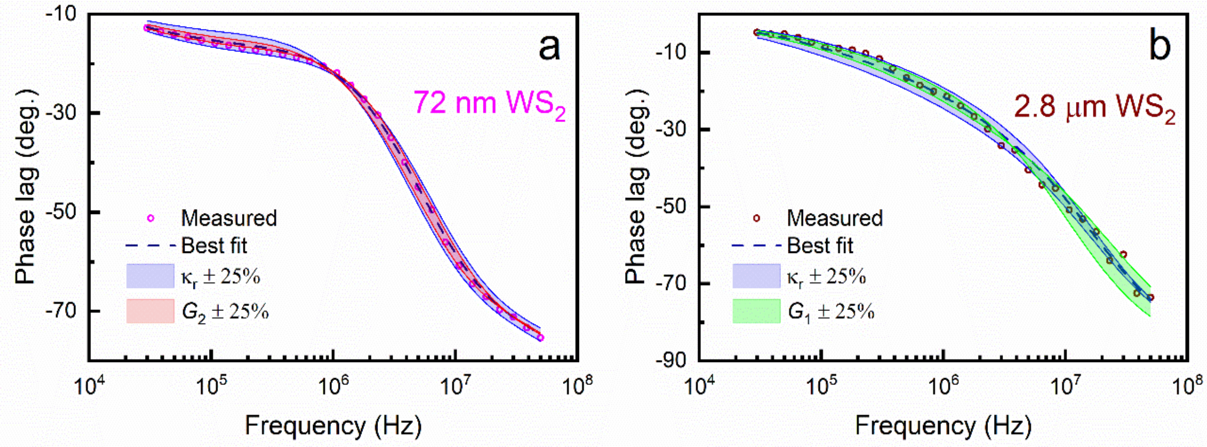

Figure S4. Conventional FDTR measurement data and fitting curves for (a) 72 nm and (b) 2.8 μm thick WS_2 flakes. The hollow symbols represent the measured phase lag and the dashed line indicates the best-fit curve. The shaded area (error band) corresponds to a ±25% variation in the fitting parameters.

In-plane thermal conductivity uncertainty

The error estimation for the in-plane thermal conductivity is much simpler compared to the out-of-plane case. This simplification arises directly from the analytical expression that links the thermal conductivity to the experimental observables. In the out-of-plane configuration, thermal conductivity can only be obtained after numerically solving an integral equation. In contrast, the in-plane case allows straightforward analytical treatment.

As shown in our recent publication by Xu *et al.*[3], the expression for the phase lag as a function of excitation frequency (*f*), spatial offset (*x*), and thermal diffusivity is given as follows, and can be readily inverted to obtain the corresponding equation for the thermal diffusivity. This inversion is the basis for propagating the experimental uncertainty:

$$\varphi(x, f^{1/2}) = x/\sqrt{(2D/\omega)} = x\sqrt{\frac{\pi f}{D}} = \sqrt{\frac{\pi}{D}}\, x f^{1/2}$$

The important aspect of the previous expression is that taking the double derivative with respect to x and $f^{1/2}$ directly yields the thermal diffusivity and, consequently, the thermal conductivity:

$$m(f) = \partial\varphi/\partial x = \sqrt{\frac{\pi}{D}} f^{1/2} \qquad \text{and,} \qquad s = \partial m(f)/\partial f^{1/2} = \sqrt{\frac{\pi}{D}}$$

$$D = \pi / s^2 \qquad \text{or,} \qquad k = (\pi / s^2)\rho C_p$$

To evaluate the error in the thermal conductivity, it is necessary to know the uncertainties in m and s, denoted as Δm and Δs. These quantities are directly obtained from the linear regression of the experimental data. Specifically, we first measure φ vs x to obtain the slopes $m(f)$ for each excitation frequency. The associated uncertainty in the regression gives Δm for each frequency. Next, we perform a linear regression of m vs $f^{1/2}$, which yields s along with its

corresponding uncertainty $\Delta s/s$. Once the errors in the single and double slopes are determined, the relative error in the thermal conductivity can be calculated by standard error propagation as:

$$\Delta k/k = 2\sqrt{[(1/N)(\Delta m/m)^2 + (\Delta s/s)^2]}$$

where N is the number of frequency points measured, assuming a negligible error in the heat capacity C_p and density ρ. To remain conservative, we also provide an upper bound for the relative error by neglecting the averaging factor $1/N$:

$$\Delta k/k = 2\sqrt{[(\Delta m/m)^2 + (\Delta s/s)^2]}$$

As an example, typical relative errors in $\Delta m/m$ are in the range of 1–2%, whereas for $\Delta s/s$ we obtain values in the range of 5–15%. Therefore, an upper bound for the relative experimental error in the thermal conductivity is typically 16%.

2.1b Determination of thermophysical properties

In addition to the fitting parameters discussed above, we incorporated other relevant thermophysical properties as input parameters in the model. As present in Figure S5 (a), the temperature-dependent specific heat capacities (C_p) of STO, gold, and WS_2 were obtained by interpolating data from the literatures.[4–6] For ease of comparison and calculation, unit conversions for literature values of specific heat capacity were carried out using the molar mass and density data provided in Table S3. Due to the low thermal expansion coefficient, the densities of all materials involved are considered constant within the chosen temperature range.[5,7,8] To determine the thermal conductivity of bulk STO substrate, we conducted variable-temperature FDTR measurements on a reference STO substrate.

Similarly, a separate silicon reference sample was used to extract the thermal conductivity of the 55 nm gold transducer via FDTR. Specifically, during the evaporation of the Au layer onto the WS_2 flakes, we simultaneously deposited an identical Au layer on a small silicon wafer that served as a reference sample. The temperature-dependent thermal conductivity of this wafer has been independently characterized using the 3-Omega method. FDTR measurements were then performed on the reference sample under identical experimental conditions, including temperature and spot size.

Figure S5 (b) shows the experimental FDTR data obtained from the 55 nm Au/Si reference sample in the temperature range 80–460 K. The solid lines represent fits to the data points, from which the cross-plane thermal conductivity of the Au transducer was extracted by simultaneously fitting the thermal conductivity of the Au layer and the thermal boundary resistance at the Au/Si interface. The thermal conductivity of the Si substrate was independently measured using the 3-Omega method, and it is shown in Figure S5 (c). Excellent agreement is found between our values and the well-established determination by Slack and Glassbrenner.[9]

Figure S5(d) presents the extracted cross-plane thermal conductivity values of the Au transducer in comparison with literature reports for Au films of similar thickness and the room temperature thermal conductivity of the Au transducer estimated using the Wiedemann–Franz law. The electrical conductivity measurements were performed by using the van der Pauw method on an independent sample consisting of an Au layer with the same thickness deposited onto a glass substrate. The results show excellent agreement with reported values,[10–13] despite the different experimental approaches employed in these studies as shown in Figure S5d.

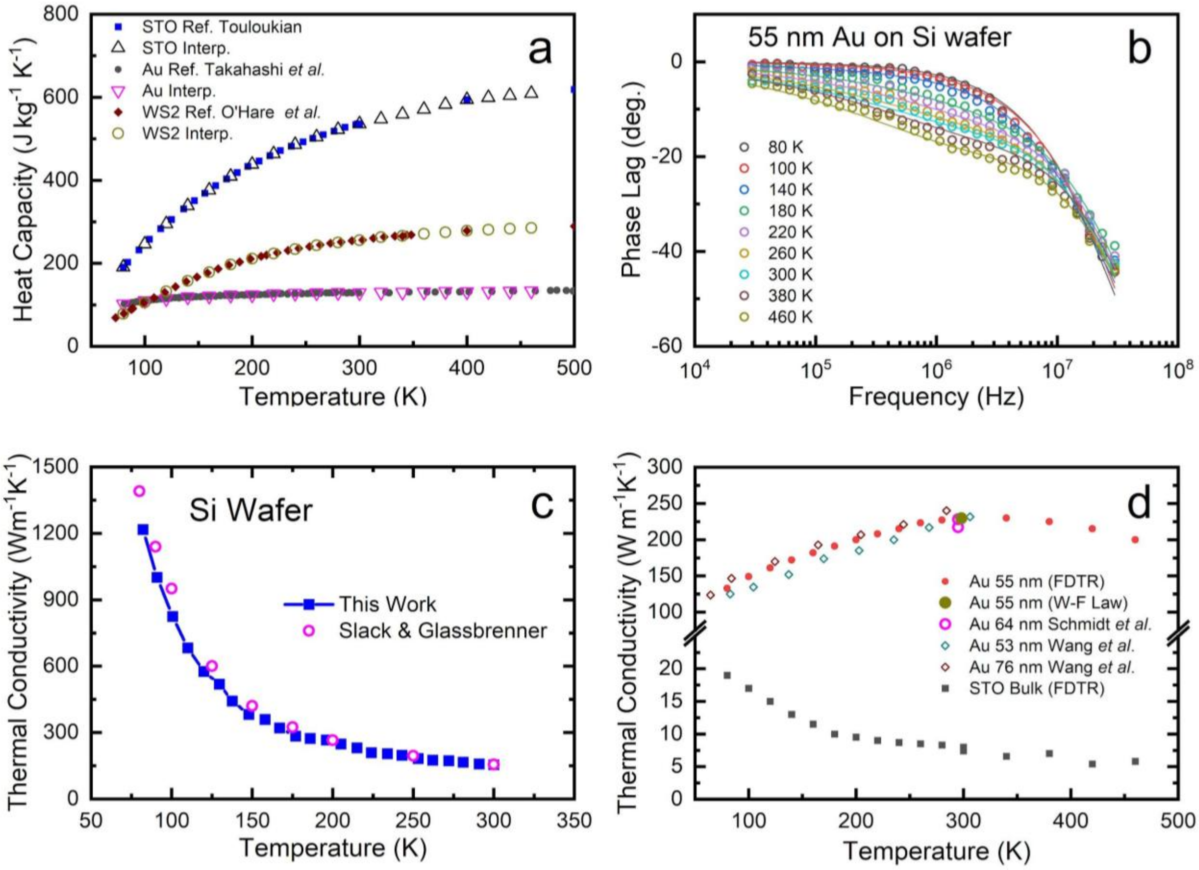

Figure S5. Temperature-dependent thermophysical properties of materials used in FDTR modelling. (a) Specific heat capacities of STO, Au, and WS_2 obtained from literatures (solid symbols) and data points obtained by interpolation (open symbols). (b) FDTR measurements on a 55 nm Au/Si reference sample from 80 to 460 K, with corresponding model fits. (c) Temperature dependent thermal conductivity of the silicon wafer measured through the 3-Omega method. (d) Thermal conductivity of bulk STO and 55 nm-thick Au transducers measured via FDTR on reference samples, shown alongside literature values and the Au transducer's thermal conductivity calculated from electrical conductivity via Wiedemann-Franz (W-F) law.

**Table S3: Set of material properties used in this work**			
Materials	Molar weight (g/mol)	Density ($kg \cdot m^{-3}$)	Linear expansively (10^{-5} K^{-1})

WS_2	247.97[6]	7500[14]	0.32[7]
STO	183.49[15]	5130[16]	3.23[5]
AU	196.97[4]	19400[17]	8.71[8]

2.2 Beam-offset FDTR to obtain in-plane thermal conductivity

2.2a Note on the influence of probe power on the BO-FDTR measurements

Regarding the potential influence of the probe laser on the measured phase lag, we would like to clarify that the probe laser's power typically represents approximately 1% of the pump laser power, corresponding to tens of microwatts. Such a low power level is insufficient to cause any measurable temperature rise, thereby ensuring it does not impact the phase lag measurements. This is achievable primarily due to the utilization of our newly developed method, which incorporates a low noise amplifier (LNA, with up to 60 dB of voltage gain, Femto GmbH) to enhance the acquired signal to the millivolt range as detailed in Xu et al.[3] This approach allows for extremely low probe laser power, thereby eliminating any potential thermal influence on the measurements.

2.2b Estimation of the thermoreflectance coefficient of WS_2 for 532 nm probe wavelength

Similar to conventional FDTR, the temperature determination of BO-FDTR also depends on the optical properties on the sample surface at the probe wavelength. Using the temperature-dependent optical constants of multilayer WS_2 reported by Munkhbat et al.[18] (see Sec. II.1, Fig. 3d), the in-plane refractive index and extinction coefficient at 532 nm are approximately $n = 4.1$ and $k = 0.8$. The Fresnel relation for normal incidence:

$$R = \frac{(n-1)^2 + k^2}{(n+1)^2 + k^2}$$

gives $R \approx 0.385 = 38.5\%$. To estimate the thermoreflectance coefficient, we linearize the dependence of R on n and k. Differentiating the Fresnel expression yields $\partial R/\partial n \approx 0.085$ and $\partial R/\partial k \approx 0.037$ at these values. Liu et al.[19] (see Figs. 3 and 5c) report typical thermo-optic coefficients of order $\partial n/\partial T 1{\sim}2 \times 10^{-4}\ K^{-1}$and $\partial k/\partial T$ up to $5 \times 10^{-4}\ K^{-1}$ near visible excitonic features. Substituting these values gives:

$$dR/dT \approx (0.085) \times (1 \times 10^{-4}) + (0.037) \times (5 \times 10^{-4}) \approx 7 \times 10^{-5}$$

This order of magnitude, $\approx 10^{-4}\,K^{-1}$, is consistent with values reported for other semiconductors in the visible range and sufficient to provide measurable reflectance modulations under FDTR conditions.

In addition, probing at 532 nm offers a distinct advantage because this wavelength lies on the low-energy flank of the B exciton in WS_2 (≈520–540 nm). As documented by Liu et al.[19] (see Fig. 5c), the B-exciton exhibits a redshift and broadening with increasing temperature, leading to a strong enhancement of $\partial k/\partial T$ and hence of $(1/R)(dR/dT)$ in this spectral region. While the absolute reflectivity of WS_2 is lower than that of a metal film, the excitonic enhancement amplifies the thermoreflectance signal and compensates for this limitation. Together with the use of lock-in detection, which provides sensitivity to reflectance changes in the 10^{-6}–10^{-7} range, these factors enabled us to obtain robust and reproducible thermoreflectance signals despite the relatively large spot sizes and modest probe powers typical of FDTR setups.

For context, the estimated thermoreflectance coefficient of multilayer WS_2 at 532 nm, $(1/R)(dR/dT) \approx 7 \times 10^{-5}\,K^{-1}$, is smaller than that of noble metals but still within the detectable range of FDTR. For example, Au films probed near 532 nm typically exhibit coefficients on the order of $(1 - 3) \times 10^{-4}\,K^{-1}$ as reported elsewhere[20] benefiting from both higher reflectivity and a larger thermo-optic response in the green spectral region. Thus, while

bare WS_2 yields a somewhat weaker thermoreflectance modulation compared to Au, the combination of its intrinsic thermo-optic coefficient, the excitonic enhancement near the B-exciton, and lock-in detection sensitivity ensures that the measured signals are well within the dynamic range of our FDTR apparatus. This comparison highlights that, although WS_2 is less optimal than Au as a transducer material, its response is nevertheless sufficiently strong for reliable extraction of thermal properties.

2.2c Beam-offset FDTR measurements with and without Au transducer

To experimentally verify the feasibility of the beam offset-FDTR measurements on the bare WS_2 surface without a gold transducer, we conducted a control experiment on a 2.8 μm-thick WS_2 flake at 80 K. A simple estimation shows that at this temperature the unit-width in-plane thermal conductance (i.e. $K^i = k^i \times d^i$, in-plane thermal conductivity multiplied by thickness) of the WS_2 flake is approximately 400 times greater than that of a 55 nm-thick gold transducer. This suggests that, from the perspective of heat transfer, the presence of the gold transducer does not significantly influence the measurement of the in-plane thermal conductivity of WS_2.

Figure S6 (a) presents the phase lag as a function of spatial offset, measured with and without the gold transducer. Due to the negative temperature coefficient of reflectivity (dR/dT) for gold at a probe wavelength of 532 nm, the phase lag of the reflected probe signal exhibits a 180-degree shift relative to the actual temperature modulation. In contrast, the dR/dT of WS_2 behaves positively. To clearly distinguish between the two cases, we directly plot the phase lag of the probe signal relative to the pump in Figure S6 (a). As expected, the phase lag for the gold transducer case is positive, while it is negative for the bare WS_2 surface. In both cases, the linear fit of the data at each frequency yields consistent slopes within the experimental error range.

As shown in Figure S6 (b), the second-level linear fit — specifically, the slope of the phase lag versus offset as a function of the square root of the excitation frequency, i.e., $\frac{\partial^2\phi}{\partial x\partial f^{1/2}}$, also demonstrates excellent agreement between the two configurations. These results confirm that reliable thermal conductivity measurements can be obtained without a gold transducer under our beam offset conditions, and the measurements were not affected by potential optical excitations introduced by the pump and probe lasers. As shown in Figures 6 (c, d**)** the slope of the linear fit ($d\phi/d\Delta x$) decreases successively with increasing temperature, indicating a reduction of the in-plane thermal conductivity at higher temperatures. Notably, for the 2.8 μm-thick WS_2 sample, the in-plane thermal conductivity increases significantly at lower temperatures. To ensure that the heat transfer model satisfies the linear approximation of the phase lag under these conditions, the measurement frequency was increased accordingly.

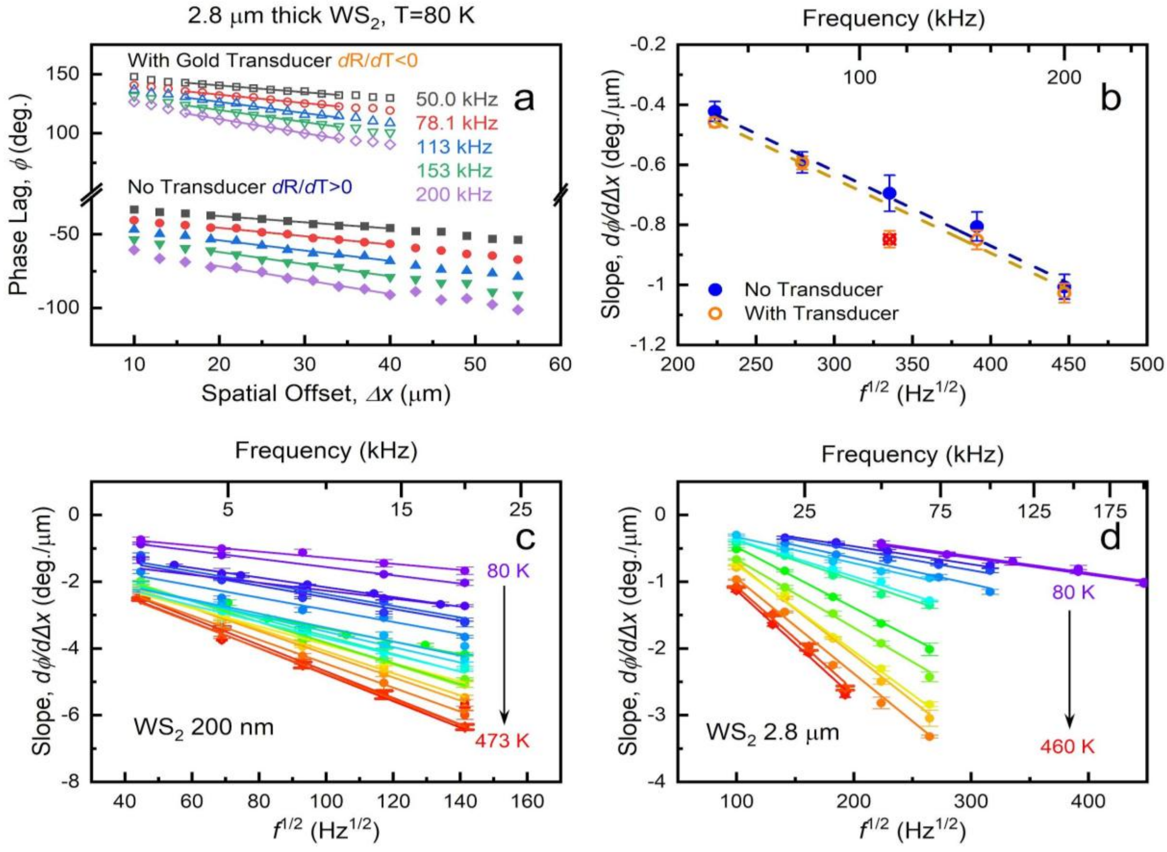

Figure S6. (a) Phase lag (ϕ) versus spatial offset (Δx) for a 2.8 μm-thick WS_2 flake at 80 K, measured across excitation frequencies ranging from 50 kHz to 200 kHz. Linear fits (solid lines) are applied to the data. Measurements were performed both with a deposited gold transducer and directly on the bare WS_2 surface. (b) Extracted slopes from the linear fits of ϕ vs. Δx plotted as a function of the square root of the excitation frequency ($f^{1/2}$). The results exhibit consistent behavior with or without the transducer within experimental error. In (c) and (d) we present the linear fit slopes ($d\phi/d\Delta x$) as a function of $f^{1/2}$ at various temperatures for WS_2 flakes with thicknesses of 200 nm and 2.8 μm, respectively. The gradients from purple to red indicate increasing temperature while the solid lines show the corresponding linear fits of the data points.

3. Computational Methods

We performed density-functional theory (DFT) calculations with the VASP code[21] and projector augmented waves[22,23] with an energy cutoff of 317 eV and the generalized-gradient approximation.[24] Van der Waals interactions are accounted for by means of the semiempirical scheme due to Grimme.[25] The Brillouin zone was sampled with a 12 × 12 × 4 k-point grid and the atomic positions were optimized until all the atomic forces were smaller than 10^{-9} eV/Å. The second- and third-order interatomic force constants (IFCs) were computed by finite differences in 6 × 6 × 2 supercells with the Phonopy[26] and the thirdorder.py[27] codes. For anharmonic IFCs, interactions up to 6.17 Å (equivalent to 8th nearest neighbors) were considered (convergence tests are shown in Figure S7). The phonon dispersion obtained with this computational setup is displayed in Figure S8.

The lattice thermal conductivity is obtained by solving the phonon Boltzmann transport equation (BTE) beyond the relaxation time approximation (RTA) on a 32 × 32 × 6 grid of **q**-points with the almaBTE code.[28] Within this approach, we properly distinguish between resistive Umklapp processes and momentum-conserving Normal processes. This allows the iterative method to capture collective (i.e., hydrodynamic) phonon transport effects that are important in 2D materials. Scattering from isotopic disorder was accounted for through the model of Tamura.[29]

We note that the data could also be interpreted using an alternative formal framework that separates the kinetic and collective contributions to thermal conductivity, known as the Kinetic-Collective Model (KCM).[30–32] The main differences between our approach (DFT+AlmaBTE) with the KCM are primarily formal. The KCM explicitly separates kinetic and collective contributions and expresses the thermal conductivity as a weighted average of these two components. While this decomposition can be useful for disentangling the role of different scattering mechanisms, it is conceptually similar to the iterative BTE approach employed here. Indeed, direct comparisons between the two methods show that they yield results in very good agreement.

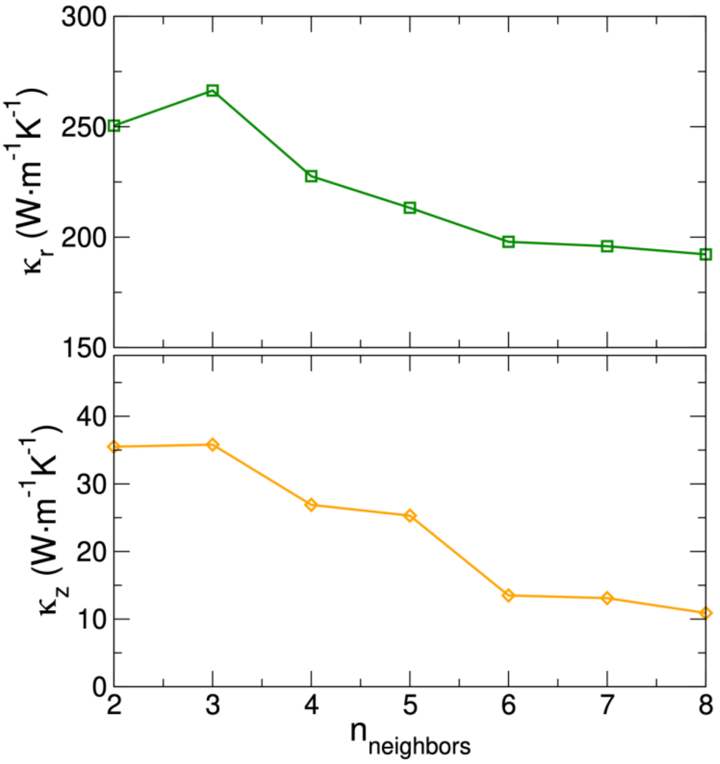

Figure S7. In-plane (top) and cross-plane (bottom) thermal conductivity of WS_2 as a function of the cutoff for third-order phonon-phonon interactions.

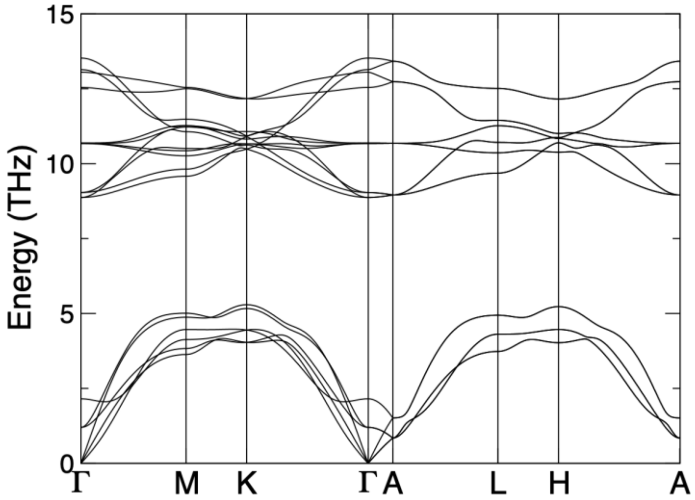

Figure S8. Phonon dispersion of bulk WS_2 from first-principles density-functional calculation

References

(1) Gao, H.; Xu, K. X.; Chen, B.; Wu, L.-Z.; Tung, C.-H.; Ji, H.-F. Ultrahydrophobicity of Polydimethylsiloxanes-Based Multilayered Thin Films. *J. Nanotechnol.* **2009**, *2009*, 1–8.

(2) Schmidt, A. J.; Cheaito, R.; Chiesa, M. A Frequency-Domain Thermoreflectance Method for the Characterization of Thermal Properties. *Review of Scientific Instruments* **2009**, *80* (9).

(3) Xu, K.; Guo, J.; Raciti, G.; Goni, A. R.; Alonso, M. I.; Borrisé, X.; Zardo, I.; Campoy-Quiles, M.; Reparaz, J. S. In-Plane Thermal Diffusivity Determination Using Beam-Offset Frequency-Domain Thermoreflectance with a One-Dimensional Optical Heat Source. *Int J Heat Mass Transf* **2023**, *214*.

(4) Takahashi, Y.; Akiyama, H. Heat Capacity of Gold from 80 to 1000 K. *Thermochim. Acta* **1986**, 109 (1), 105–109.

(5) de Ligny, D.; Richet, P. High-Temperature Heat Capacity and Thermal Expansion of and Perovskites. *Phys. Rev. B* **1996**, 53 (6), 3013 3022.

(6) O'Hare, P. A. G.; Hubbard, W. N.; Johnson, G. K.; Flotow, H. E. Calorimetric Measurements of the Low-Temperature Heat Capacity, Standard Molar Enthalpy of Formation at 298.15 K, and High-Temperature Molar Enthalpy Increments Relative to 298.15 K of Tungsten Disulfide (WS2), and the Thermodynamic Properties to 1500 K. *J. Chem. Thermodyn*. **1984**, 16 (1), 45–59.

(7) Hu, X.; Yasaei, P.; Jokisaari, J.; Öğüt, S.; Salehi-Khojin, A.; Klie, R. F. Mapping Thermal Expansion Coefficients in Freestanding 2D Materials at the Nanometer Scale. *Phys. Rev. Lett.* **2018**, *120* (5), 1–6.

(8) Oliva, A. I.; Lugo, J. M.; Gurubel-Gonzalez, R. A.; Centeno, R. J.; Corona, J. E.; Avilés, F. Temperature Coefficient of Resistance and Thermal Expansion Coefficient of 10-Nm Thick Gold Films. *Thin Solid Films* **2017**, *623*, 84–89.

(9) Glassbrenner, C. J.; Slack, G. A. Thermal Conductivity of Silicon and Germanium from 3°K to the Melting Point. *Phys. Rev.* **1964**, *134*, A1058–A1069.

(10) Schmidt, A. J.; Cheaito, R.; Chiesa, M. Characterization of Thin Metal Films via Frequency-Domain Thermoreflectance. *J. Appl. Phys.* **2010**, *107* (2).

(11) Wang, H. D.; Liu, J. H.; Zhang, X.; Guo, Z. Y.; Takahashi, K. Experimental Study on the Influences of Grain Boundary Scattering on the Charge and Heat Transport in Gold and Platinum Nanofilms. *Heat and Mass Transf.* **2011**, *47* (8), 893–898.

(12) Wang, H.; Liu, J.; Zhang, X.; Takahashi, K. Breakdown of Wiedemann-Franz Law in Individual Suspended Polycrystalline Gold Nanofilms down to 3 K. *Int J Heat Mass Transf.* **2013**, *66*, 585–591.

(13) Yu, C.; Scullin, M. L.; Huijben, M.; Ramesh, R.; Majumdar, A. Thermal Conductivity Reduction in Oxygen-Deficient Strontium Titanates. *Appl Phys Lett* **2008**, *92* (19).

(14) Yakushchenko, S. V.; Brailo, M. V.; Sapronov, O. O.; Zinchenko, S. M. Study of Dispersed Additives for the Formation of Polymer Composite Materials to Increase the Performance Characteristics of Friction Units of Transport Vehicles. *J. Hydrocarb. Power Eng.* **2022**, *9* (1), 1–7.

(15) Chen, B. R.; Crosby, L. A.; George, C.; Kennedy, R. M.; Schweitzer, N. M.; Wen, J.; Van Duyne, R. P.; Stair, P. C.; Poeppelmeier, K. R.; Marks, L. D.; Bedzyk, M. J. Morphology and CO Oxidation Activity of Pd Nanoparticles on SrTiO3 Nanopolyhedra. *ACS Catal.* **2018**, *8* (6), 4751–4760.

(16) Wang, Y.; Fujinami, K.; Zhang, R.; Wan, C.; Wang, N.; Ba, Y.; Koumoto, K. Interfacial Thermal Resistance and Thermal Conductivity in Nanograined SrTiO3. *Applied Physics Express* **2010**, *3* (3).

(17) Tanimoto, H.; Sakai, S.; Kita, E.; Mizubayashi, H. Characterization and Determination of Elastic Property of High-Density Nanocrystalline Gold Prepared by Gas-Deposition Method. *Mater. Trans.* **2003**, 44 (1), 94–103.

(18) Munkhbat, B.; Wróbel, P.; Antosiewicz, T. J.; Shegai, T. O. Optical Constants of Several Multilayer Transition Metal Dichalcogenides Measured by Spectroscopic Ellipsometry in the 300-1700 Nm Range: High Index, Anisotropy, and Hyperbolicity. *ACS Photonics* **2022**, *9* (7), 2398–2407.

(19) Liu, H. L.; Yang, T.; Chen, J. H.; Chen, H. W.; Guo, H.; Saito, R.; Li, M. Y.; Li, L. J. Temperature-Dependent Optical Constants of Monolayer MoS2 , MoSe2 , WS2 , and WSe2 : Spectroscopic Ellipsometry and First-Principles Calculations. *Sci. Rep.* **2020**, *10* (1).

(20) Paddock, C. A.; Eesley, G. L. Transient Thermoreflectance from Thin Metal Films. *J Appl Phys* **1986**, *60* (1), 285–290.

(21) Kresse, G.; Furthmüller, J. Efficient Iterative Schemes for Ab Initio Total-Energy Calculations Using a Plane-Wave Basis Set. *Phys. Rev. B* **1996**, *54* (16), 11169–11186.

(22) Blöchl, P. E. Projector Augmented-Wave Method. *Phys. Rev. B* **1994**, 50 (24), 17953 17979.

(23) Joubert, D. From Ultrasoft Pseudopotentials to the Projector Augmented-Wave Method. *Phys Rev B Condens Matter Mater Phys* **1999**, *59* (3), 1758–1775.

(24) Perdew, J. P.; Burke, K.; Ernzerhof, M. Generalized Gradient Approximation Made Simple. Phys. Rev. Lett. **1996**, 77 (18), 3865–3868.

(25) Grimme, S. Semiempirical GGA-Type Density Functional Constructed with a Long-Range Dispersion Correction. *J. Comput. Chem.* **2006**, *27* (15), 1787–1799.

(26) Togo, A.; Tanaka, I. First Principles Phonon Calculations in Materials Science. *Scr. Mater.* **2015**, *108*, 1–5.

(27) Li, W.; Carrete, J.; Katcho, N. A.; Mingo, N. ShengBTE: A Solver of the Boltzmann Transport Equation for Phonons. *Comput. Phys. Commun.* **2014**, *185* (6), 1747–1758. .

(28) Carrete, J.; Vermeersch, B.; Katre, A.; van Roekeghem, A.; Wang, T.; Madsen, G. K. H.; Mingo, N. AlmaBTE: A Solver of the Space-Time Dependent Boltzmann Transport Equation for Phonons in Structured Materials. *Comput. Phys. Commun.* **2017**, *220*, 351–362.

(29) Tamura, S.-I. Isotope Scattering of Dispersive Phonons in Ge. *Phys. Rev. B* **1983**, *27* (2), 858–866

(30) De Tomas, C.; Cantarero, A.; Lopeandia, A. F.; Alvarez, F. X. From Kinetic to Collective Behavior in Thermal Transport on Semiconductors and Semiconductor Nanostructures. *J. Appl. Phys.* **2014**, *115* (16).

(31) Torres, P.; Torelló, A.; Bafaluy, J.; Camacho, J.; Cartoixà, X.; Alvarez, F. X. First Principles Kinetic-Collective Thermal Conductivity of Semiconductors. *Phys. Rev. B* **2017**, *95* (16)

(32) Torres, P.; Alvarez, F. X.; Cartoixà, X.; Rurali, R. Thermal Conductivity and Phonon Hydrodynamics in Transition Metal Dichalcogenides from First-Principles. *2D Mater.* **2019**, *6* (3).
